# Rab1 interacts with GOLPH3 and controls Golgi structure and contractile ring constriction during cytokinesis in *Drosophila melanogaster*

**DOI:** 10.1098/rsob.160257

**Published:** 2017-01-18

**Authors:** Stefano Sechi, Anna Frappaolo, Roberta Fraschini, Luisa Capalbo, Marco Gottardo, Giorgio Belloni, David M. Glover, Alan Wainman, Maria Grazia Giansanti

**Affiliations:** 1 Istituto di Biologia e Patologia Molecolari del CNR, Dipartimento di Biologia e Biotecnologie, Università Sapienza di Roma, Piazzale A. Moro 5, 00185 Roma, Italy; 2 Dipartimento di Biotecnologie e Bioscienze, Università degli studi di Milano Bicocca, Piazza della Scienza 2, 20126 Milan, Italy; 3 Department of Genetics, University of Cambridge, Downing Street, Cambridge CB2 3EH, UK; 4 Dipartimento di Scienze della Vita, Università di Siena, Via A. Moro 2, 53100 Siena, Italy; 5 Sir William Dunn School of Pathology, University of Oxford, South Parks Road, Oxford OX1 3RE, UK

**Keywords:** *Drosophila*, cytokinesis, Rab1, Golgi

## Abstract

Cytokinesis requires a tight coordination between actomyosin ring constriction and new membrane addition along the ingressing cleavage furrow. However, the molecular mechanisms underlying vesicle trafficking to the equatorial site and how this process is coupled with the dynamics of the contractile apparatus are poorly defined. Here we provide evidence for the requirement of Rab1 during cleavage furrow ingression in cytokinesis. We demonstrate that the gene *omelette* (*omt*) encodes the *Drosophila* orthologue of human Rab1 and is required for successful cytokinesis in both mitotic and meiotic dividing cells of *Drosophila melanogaster*. We show that Rab1 protein colocalizes with the conserved oligomeric Golgi (COG) complex Cog7 subunit and the phosphatidylinositol 4-phosphate effector GOLPH3 at the Golgi stacks. Analysis by transmission electron microscopy and 3D-SIM super-resolution microscopy reveals loss of normal Golgi architecture in *omt* mutant spermatocytes indicating a role for Rab1 in Golgi formation. In dividing cells, Rab1 enables stabilization and contraction of actomyosin rings. We further demonstrate that GTP-bound Rab1 directly interacts with GOLPH3 and controls its localization at the Golgi and at the cleavage site*.* We propose that Rab1, by associating with GOLPH3, controls membrane trafficking and contractile ring constriction during cytokinesis.

## 1. Background

Cytokinesis represents the final act of cell division when a mother cell becomes fully partitioned into two daughter cells [1]. Cytokinesis failures can contribute to several human diseases including blood disorders [2], age-related macular degeneration [3], Lowe syndrome [4], female infertility [1,5] and cancer [1,5]. In animal cells, cytokinesis relies upon constriction of a plasma membrane-anchored actomyosin ring, which leads to cleavage furrow ingression at the equatorial cortex [1]. To fully separate each mother cell into two daughter cells, cytokinesis is also associated with a considerable expansion of cell plasma membrane [1]. Insertion of new membrane during cytokinesis is achieved through shuttling of membrane vesicles to the ingressing cleavage furrow and involves both secretory and endocytic/recycling trafficking activities [1,6]. Accumulating evidence also indicates that phosphoinositide lipids regulate both contractile ring dynamics and membrane trafficking during cytokinesis [7]. *Drosophila* male meiosis provides an excellent cell system to dissect the vesicle trafficking pathways involved in cytokinesis [8,9]. Indeed screens for mutants affecting spermatocyte cytokinesis have identified several components of the Golgi and endocytic/recycling machinery, comprising the conserved oligomeric Golgi complex (COG) subunits Cog5 and Cog7, the TRAPPII complex subunit Brunelleschi, the syntaxin 5 ER-to-Golgi vesicle-docking protein, the small GTPases Rab11 and Arf6, the COPI subunits and the exocyst complex proteins Sec8 and Exo84 [10–17]. Mutations affecting male meiotic cytokinesis have also revealed the requirement for proteins that regulate the phosphoinositide pathway including the *Drosophila* phosphatidylinositol (PI) transfer protein (PITP) Giotto/Vibrator (Gio/Vib) and the PI 4-kinase III β Four wheel drive (Fwd) [18–20]. Both Fwd and Gio/Vib are required to localize Rab11 at the cleavage site [18,21]. Fwd directly binds Rab11 at the Golgi and is required for synthesis of PI 4-phosphate (PI(4)P) on Golgi membranes and for localization of secretory organelles containing both PI(4)P and Rab11 at the cleavage site [21]. We have recently shown that the oncoprotein GOLPH3, described as a PI(4)P effector at the Golgi [22], accumulates at the cell equator of dividing cells and is required for cleavage furrow ingression in *Drosophila* [23]. GOLPH3 function during cytokinesis is intimately connected to its ability to bind PI(4)P and regulates both the dynamics of the actomyosin ring and vesicle trafficking to the cleavage site [22–24].

The small GTPase Rab1 regulates endoplasmic reticulum (ER) to Golgi and intra-Golgi trafficking through different effectors [25,26]. Rab1, in its GTP-bound, active form, binds the tethering factors p115 [27] and GM130 [28,29] which regulate coat protein II (COPII) mediated ER-to-Golgi transport. Rab1 also modulates coat protein I (COPI) recruitment by binding the GBF-type (Golgi-brefeldin A resistance factor) ADP-ribosylation factor guanine nucleotide exchange (ARFGEF) factor [30]. Rab1 proteins have been involved in several cellular signalling pathways that include nutrient signalling [31,32], Notch signalling [33], cell migration [34] and regulation of autophagy [35,36]. Moreover, deregulation of *Rab1* expression has been linked to several human cancer types [31,32,37–40] and other human diseases including cardiomyopathy [41] and Parkinson's disease [42,43]. Recent work has suggested that a complex of human Rab1B with the oncogene PITPNC1, by augmenting PI(4)P Golgi levels, might indirectly enhance recruitment of GOLPH3 to the Golgi and facilitate Golgi extension and vesicular secretion of pro-tumour factors in cancer cells [44]. Here we provide the first evidence for a role of Rab1 in cytokinesis. We show that the gene *omelette*, identified during a screen for mutants affecting male meiotic cytokinesis [45], encodes the *Drosophila* orthologue of human Rab1 and is required for contractile ring constriction during cytokinesis of both mitotic and meiotic cells. We demonstrate that Rab1 directly interacts with GOLPH3 and contributes to the architecture of interphase Golgi stacks in *Drosophila* spermatocytes. We further show that Rab1 enables localization of the GOLPH3 complex at the cleavage furrow. We propose that Rab1, by recruiting GOLPH3 at the Golgi membranes, controls the flow of secretory vesicle trafficking that is necessary for proper furrow ingression during cytokinesis.

## 2. Results

### 2.1. The *Drosophila* homologue of Rab1, *omelette*, is required for cytokinesis during meiosis

**Figure 1.**
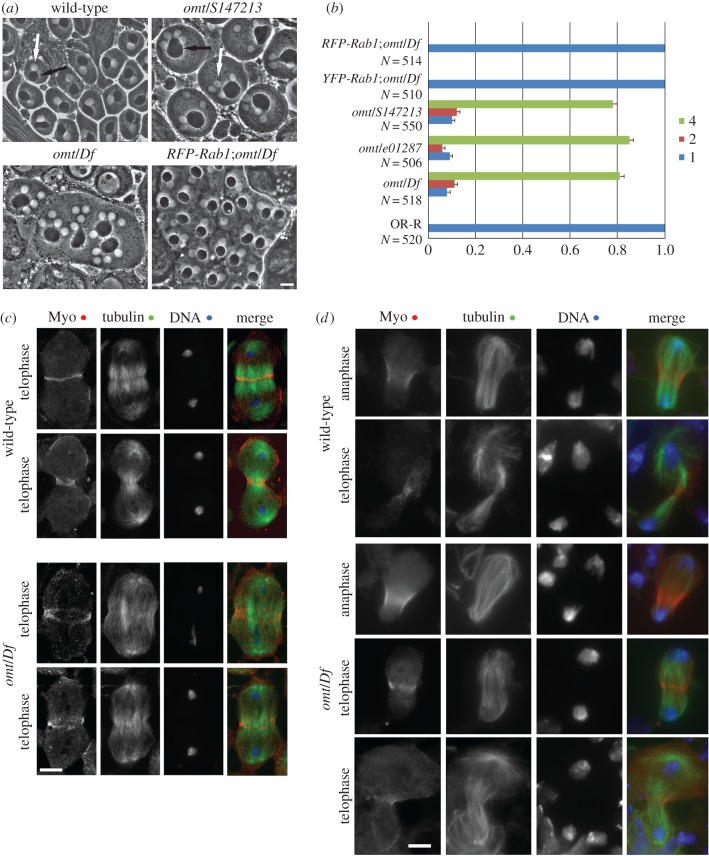
Rab1 is required for cytokinesis in meiotic and mitotic cells. (*a*) Rescue of *omt* cytokinesis defects by Rab1. Testes from 3–5 days old males carrying either *omt^z4144^/Df(3R)ED10338 (omt/Df)* or *omt^z4144^*/*S147213 (omt/S147213)* genotypes, viewed using phase-contrast microscopy, display irregular spermatids containing multiple nuclei (phase light, white arrow) associated with enlarged mitochondrial derivatives (nebenkern, phase dark, black arrow). Each wild-type spermatid displays a single nucleus (white arrow) associated with a nebenkern (black arrow) of similar size. A single copy of RFP-Rab1 transgene can rescue the cytokinesis defects associated with *omt/Df* mutation. A minimum of 500 spermatids, derived from at least 10 males, was examined for each genotype. Scale bar, 10 µm. (*b*) Frequencies of spermatids containing 1, 2 or 4 nuclei per mitochondrial derivative in testes from *omt* mutants (*omt^z4144^/Df(3R)ED10338 (omt/Df)* mutants, *omt/S147213*, *omt^z4144^*/*Rab1^e01287^*(*omt/e01287*)) in wild-type and in testes from males of genotype *RFP-Rab1*;*omt/Df* or *YFP-Rab1*;*omt/Df.* Error bars indicate s.e.m. values. (*c*) Wild-type and *omt^z4144^/Df(3R)ED10338 (omt/Df)* mutant spermatocytes during early telophase and mid–late telophase stained for the myosin II heavy chain Zipper (Myo), tubulin and DNA. In total, *N* = 33 wild-type mid–late telophase cells and *N* = 28 *omt*/*Df* mutant mid–late telophase cells were analysed. The cells examined were from preparations of individual testes in three independent experiments. Scale bar, 10 µm. (*d*) Wild-type and *omt/Df* larval neuroblasts during anaphase/early telophase (anaphase) and late telophase (telophase) stained for Zipper, tubulin and DNA. In total, *N* = 176 wild-type mid–telophase cells and *N* = 180 *omt/Df* mutant mid-telophase cells were analysed. Mid–late telophase cells were examined from eight preparations of individual larval brains per each genotype; cells were examined in three independent experiments. Scale bar, 5 µm.

The *omelette^z4144^* (*omt^z4144^*) allele was identified during a screen for mutations that disrupt cytokinesis in *Drosophila* spermatocytes [45]. The *omt^z4144^* mutation was mapped to a single interval, between *stripe* and *claret* on the third chromosome [45]. The interval was further delineated to the chromosomal region 93C6–93E1, defined by the deletion *Df(3R)e^F1^*, which failed to complement *omt^z4144^* [45]. Complementation analysis with a series of chromosomal deletions uncovering the interval 93C6–93E1, revealed that *omt^z4144^* complemented *Df(3R)GC14*, but failed to complement both *Df(3R)ED10845* and *Df(3R)ED10838* for the male sterility and male meiotic defects, indicating that it maps to a region that contains the annotated *CG3320* gene (figure 1*a*,*b*; electronic supplementary material, figure S1*a*). *CG3320* encodes a polypeptide of 205 amino acids that is 82.9% identical to human Rab1A and 82.1% to human Rab1B [46] (electronic supplementary material, figure S1*b*). Thus, hereafter we refer to *CG3320* as *Drosophila Rab1.* Two P-element lethal insertions in *Rab1*, namely *Rab1^S147213^* and *Rab1^e01287^*, failed to complement *omt^z4144^* for both the male sterility and meiotic cytokinesis phenotype indicating that *omt^z4144^* is a mutant allele of *Rab1* (figure 1*a*,*b*; electronic supplementary material, figure S1*a*). YFP-Rab1 protein expressed under the control of the male germ line promoter *spermatocyte arrest* (*sa*, [47]) and RFP-Rab1 expressed under the control of a tubulin promoter fully rescued the cytokinesis defects of *omt^z4144^*/*Df(3R)ED10838 (omt*/*Df)* mutant males, confirming that the cytokinesis phenotype is the consequence of a mutation in the *Rab1* locus (figure 1*a*,*b*). Sequencing of the *Rab1* gene in the EMS-induced *omt^z4144^* mutants, failed to reveal alterations in the protein coding exons when compared with the DNA sequence of the original Zuker-background chromosome. However, as western blot analysis indicated that Rab1 expression was strongly reduced in *omt*/*Df* and *omt^z4144^*/*Rab1^S147213^* mutants (see below), we surmise that the molecular lesion in the *omt^z4144^* mutant allele is likely to affect some regulatory elements. Our previous characterization of *omt* mutants suggested that F-actin and anillin rings formed normally during early stages of telophase but appeared broken or unconstricted in late telophase [45]. To gain further insight into the cytokinesis phenotype of the *omt*/*Df* mutant, telophase spermatocytes were stained for the myosin II heavy chain Zipper ([48], figure 1*c*). All spermatocytes from both wild-type and *omt*/*Df* mutant males assembled Zipper rings at the cell equator during early stages of telophase (figure 1*c*). However, during later stages of cytokinesis, 100% of mid-telophases from wild-type cells displayed constricted Zipper rings (figure 1*c*), whereas 75% of mid–late telophases from *omt*/*Df* mutants displayed unconstricted and fragmented Zipper rings (*N* = 33 wild-type mid–late telophase cells; *N* = 28 *omt*/*Df* mutant mid–late telophase cells). Localization of Pavarotti (Pav) [49], the *Drosophila* orthologue of human MKLP1, was also affected in *omt/Df* mutant spermatocytes. Wild-type spermatocytes at mid-telophase displayed a tight equatorial band of Pav (100% of dividing mid-telophases, *N* = 48). Conversely, 75% of mid-telophases from *omt/Df* mutant spermatocytes (*N* = 44) displayed only weak concentration of Pav at both peripheral and interior microtubules (electronic supplementary material, figure S2*a*).

### 2.2. *Drosophila* Rab1 is required for cytokinesis in mitotic cells

**Figure 2.**
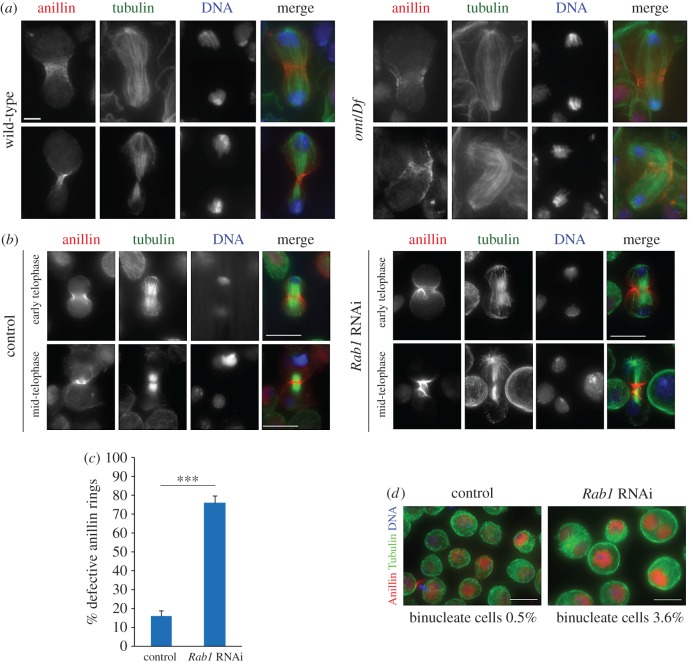
Rab1 is required for cytokinesis in larval neuroblasts and S2 cells. (*a*) Wild-type and *omt/Df* larval neuroblasts during anaphase/early telophase (upper panels) and mid–late telophase stained for anillin, α-tubulin and DNA. In 100% of wild-type cells, anillin rings appear constricted during mid–late telophase. By contrast, 31% of dividing neuroblasts from the *omt/Df* mutants displayed large and broken anillin rings. In total, *N* = 48 wild-type mid-telophases and *N* = 52 mid-telophases from *omt/Df* mutants were analysed from preparations of six individual larval brains per each genotype; cells were examined in three independent experiments. Scale bar, 5 µm. (*b*) Rab1 depletion impairs anillin localization in cytokinesis. Cells were treated with Rab1 dsRNA (*Rab1* RNAi) or with kanamycin dsRNA (control) for 72 h and then fixed and stained to reveal anillin, tubulin and DNA. (*c*) Quantification of defective anillin rings in S2 cells treated with Rab1 dsRNA (*Rab1* RNAi) or with kanamycin dsRNA (control) for 72 h. ***p < 0.0001. (*d*) S2 cells fixed and stained to detect anillin, tubulin and DNA. The percentage of binucleate cells was calculated using the results of three independent experiments. More than 2000 cells were counted in each experiment. Scale bar, 10 µm.

The gene *Rab1* is essential for normal development and viability in *Drosophila*; animals of genotypes *Rab1^S147213^/Rab1^S147213^*, *Rab1^e01287^/Rab1^e01287^* or *Rab1^S147213^/Rab1^01287^* die during early larval stages (electronic supplementary material, figure S1*a*) and adult flies of genotype *omt*/*Df* have a reduced lifespan when compared with control siblings (only 2% of *omt*/*Df* animals (*N* = 300) survive after ten days from the eclosion, compared with 100% of wild-type animals (*N* = 280)). A single copy of the RFP-Rab1 transgene rescued the semi-lethality of *omt*/*Df* animals confirming that this phenotype is due to a mutation in *Rab1*. Immunostaining of larval brains for tubulin and either Zipper or anillin revealed cytokinesis defects in dividing neuroblasts (figures 1*d* and 2*a*). One hundred per cent of mid-telophases from wild-type larval neuroblasts (*N* = 176 mid-telophases) displayed constricted Zipper rings (figure 1*c*) whereas 30% of mid-telophases (*N* = 180 mid-telophases) from *omt*/*Df* mutants displayed large and unconstricted Zipper rings, indicating the requirement for Rab1 for mitotic cytokinesis of larval neuroblasts. Similarly, all the anillin rings from wild–type larval neuroblasts appeared constricted during mid-late telophase (100% of mid-telophases, *N* = 48). By contrast, 31% of dividing neuroblasts from the *omt/Df* mutants displayed large and broken anillin rings (*N* = 52 mid-telophases). We also used RNAi to knock down Rab1 GTPase in *Drosophila* S2 cells. We found unconstricted anillin rings at the cleavage furrow after depletion of Rab1 (figure 2*b*,*c*) similar to the defective anillin rings of larval brain neuroblasts from *omt/Df* mutant animals (figure 2*a).* Depletion of Rab1 GTPase in dividing S2 DMel cells also impaired formation of tight Pav bands at the cell equator in mid-telophases (electronic supplementary material, figure S2*b*). Consistent with a role for Rab1 in cytokinesis, S2 cells treated with double-stranded RNA against Rab1 displayed a significant increase in the number of binucleate cells compared with control cells (*N* = 2000 cells from three independent experiments (figure 2*d*).

Taken together these results indicate that Rab1 is required for cytokinesis in mitotic dividing cells.

### 2.3. Rab1 localizes to Golgi organelles and is enriched at the cleavage site during telophase

**Figure 3.**
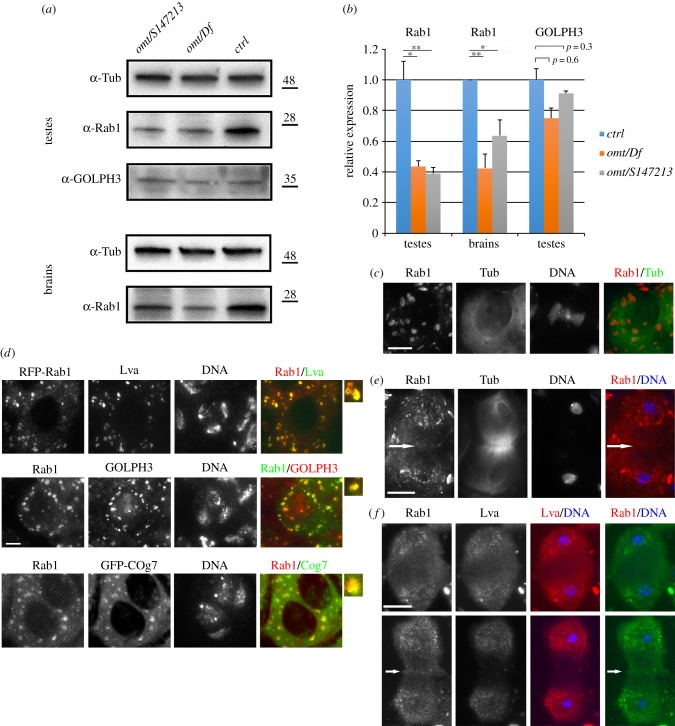
Rab1 localizes to Golgi organelles and concentrates at the cleavage site during telophase. (*a*) Western blot from adult testis (testes) or larval brains extracts (brains). Polyclonal mouse anti-Rab1 (S12085a) antibodies against *Drosophila* Rab1 recognized a band of 23 kDa that is strongly reduced in extracts from *omt/Df* and *omt/S147213* mutants. α-Tubulin (Tub) was used as a loading control. Western blots were also probed with rabbit anti-GOLPH3 (G49139/77) antibodies to analyse GOLPH3 expression levels. (*b*) Quantification of the expression levels of Rab1 and GOLPH3 proteins in western blots from adult testis (testes) or larval brains extracts (brains). Band intensities from three independent experiments were quantified using Image Lab software. The intensity of each band relative to the intensity of loading control (tubulin), was normalized to the wild-type control. Error bars indicate s.e.m. Statistically differences are *p < 0.05; **p < 0.01. (*c*) Interphase spermatocytes stained for tubulin (Tub), Rab1 and DNA. (*d*) Colocalization of Rab1 with the Golgi proteins Lva, GOLPH3 and Cog7 in interphase spermatocytes. Enlarged panels show colocalization of the proteins at the Golgi. At least *N* = 30 interphase spermatocytes were examined for each double staining. The cells examined for Golgi analysis were randomly selected from images taken in four independent experiments. (*e*) Dividing spermatocytes during telophase stained for Rab1, α-tubulin (Tub) and DNA. *N* = 60 dividing spermatocytes were examined from images taken in five independent experiments. (*f*) Anaphase (upper panel) and telophase (lower panel) spermatocytes stained for Rab1, Lva and DNA. *N* = 26 anaphases and *N* = 32 telophases were examined in testes stained for Rab1 and Lva. Dividing spermatocytes were examined from images taken in three independent experiments. Arrows in (*e*) and (*f*) point to Rab1 enrichment at the cleavage site. Scale bars, 10 µm.

To analyse the subcellular localization of Rab1, we raised polyclonal antibodies against *Drosophila* Rab1 protein that recognized a band of the predicted molecular weight in western blots from extracts of adult testes and larval brains (figure 3*a*; electronic supplementary material, figure S3*a*). The intensity of the Rab1 band appeared strongly reduced in testis and brain extracts from *omt*/*Df* and *omt^z4144^*/*Rab1^S147213^* mutants, compared with wild-type, indicating that the antibodies specifically reacted with *Drosophila* Rab1 (figure 3*a*,*b*). Indeed both mouse and rabbit anti-Rab1 antibodies recognized a 23 kDa band that appeared significantly reduced in S2 DMel cells that were depleted of Rab1, thus confirming the specific antigen binding of these antibodies (electronic supplementary material, figure S3*b*,*c*). Immunofluorescence analysis of interphase spermatocytes revealed that Rab1 protein localized to multiple structures (electronic supplementary material, figure S3*d*) that also contain the Golgi proteins Lava lamp (Lva) [50], GOLPH3 and Cog7 (figure 3*c*,*d*). In dividing spermatocytes, from metaphase to telophase, Rab1 was associated with Golgi organelles in the polar regions of the cell (figure 3*e*,*f*). During telophase the majority of Rab1 protein was enriched at the polar regions of the cell, in a pattern comparable with Lva (figure 3*e*,*f*). However, unlike Lva, Rab1 also concentrated at the cleavage furrow of dividing spermatocytes during telophase (figure 3*e*,*f*; electronic supplementary material, figure S4). As in spermatocytes, Rab1 was also visualized at the cleavage furrow of normal S2 DMel cells but not in cells that were treated with double-stranded RNA against Rab1 (electronic supplementary material, figure S3*e*).

### 2.4. Mutations in *Drosophila Rab1* affect the architecture of Golgi stacks in premeiotic primary spermatocytes

**Figure 4.**
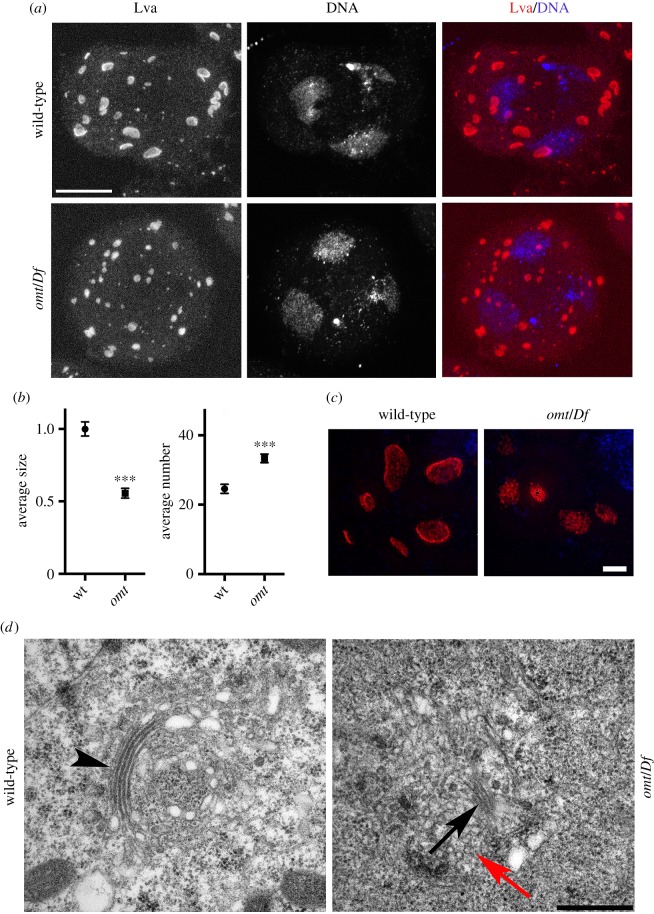
Mutations in *Rab1* disrupt Golgi architecture in premeiotic primary spermatocytes. (*a*,*c*) Interphase spermatocytes, stained for DNA and Lva. Scale bar, 10 µm. A total of *N* = 31 cells from wild-type and *N* = 29 cells from *omt*/*Df* were examined*.* Cells were imaged in six independent experiments. (*b*) Average size (relative to wild-type ± s.e.m.) and average number (±s.e.m.) of Lva-positive bodies quantified using the ImageJ software, in wild-type and *omt*/*Df (omt)* mutant males. Statistically significant differences are ***p < 0.0001 (for Golgi number, *p* = 5.39979 × 10^−6^; for Golgi size, *p* = 4.70466 × 10^−10^). In total, *N* = 761 Golgi were examined in wild-type and *N* = 968 in *omt*/*Df* mutant spermatocytes. The cells examined for Golgi analysis were randomly selected from images taken in six independent experiments. (*c*) Representative images of Golgi stacks visualized with anti-Lva (red) using 3D-SIM super-resolution microscopy. Scale bar, 2 µm. (*d*) Transmission electron micrographs showing details of Golgi stacks in primary spermatocytes. Wild-type spermatocytes display distinct stacks (arrowhead), whereas reduced stacks are visible in mutant germ cells (black arrow). Red arrow points to vesicles. Scale bar, 500 nm.

Several *Drosophila* genes encoding membrane-trafficking proteins are required for the proper structure of Golgi stacks in interphase primary spermatocytes [11,17,23]. Rab1 is essential for maintaining Golgi structure in *Drosophila* spermatocytes, consistent with the previous finding that human Rab1 controls Golgi architecture [51,52]. In premeiotic wild-type spermatocytes at stage S5 [23], stained for the golgin Lva, the average number of fluorescent bodies per cell was 25 (*N* = 31 cells from six independent experiments) (figure 4*a*,*b*). Conversely, spermatocytes from *omt*/*Df* mutant males, stained for Lva at the same stage, exhibited a 1.4-fold increase in the number of fluorescent bodies (average of 33 (*N* = 29 cells examined, from six independent experiments)) (figure 4*a*,*b*), with the average size decreased by 55% (figure 4*a*,*b*)*.* We further investigated the change in architecture of the Golgi in *omt/Df* mutants using 3D-SIM super-resolution microscopy. The increased resolution revealed that the Golgi stacks appear ‘collapsed’ in *omt/Df* mutant cells (figure 4*c*). Interphase spermatocytes from *omt*/*Df* mutant males, analysed by transmission electron microscopy (TEM), displayed abnormal Golgi complexes, comprising fragmented cisternae and a few small stacks (15 of 17 Golgi examined; figure 4*d*). By contrast, in wild-type primary spermatocytes, Golgi complexes comprised several cisternae assembled in larger stacks (15 of 15). Moreover, the Golgi bodies of *omt*/*Df* mutant spermatocytes exhibited a large number of associated small vesicles (15 of 17; figure 4*d*) suggesting defects in vesicle targeting to Golgi membranes.

### 2.5. Relationship between Rab1, GOLPH3 and Cog7 proteins at the Golgi membranes

**Figure 5.**
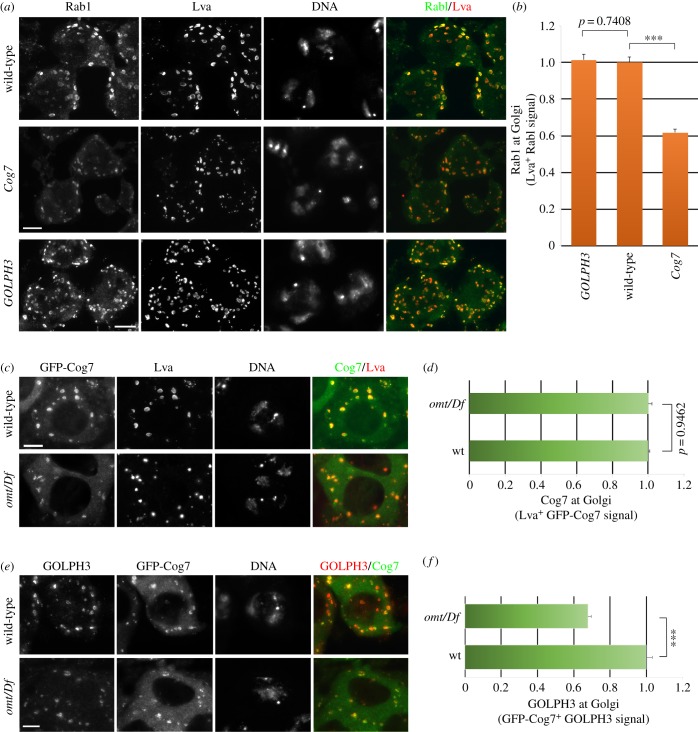
Relationship between Rab1, GOLPH3 and Cog7 proteins, at the Golgi stacks of interphase spermatocytes. (*a*) Interphase spermatocytes from wild-type, *Cog7^z4495^/Df(3R)BSC861* (*Cog7*) and *sau^z2217^*/*Df(2L)Exel7010 (GOLPH3)* mutants were stained for Rab1, Lva and DNA. (*b*) Rab1 levels in the Golgi, quantified as mean fluorescence intensity of Rab1, in Lva-positive regions (Lva^+^) (see Material and methods). In total, we examined *N* = 80 Golgi from *Cog7^z4495^/Df(3R)BSC861* (*Cog7*), *N* = 91 Golgi from *sau^z2217^*/*Df(2L)Exel7010 (GOLPH3)* mutant interphase spermatocytes and *N* = 100 Golgi in wild-type interphase spermatocytes*.* The cells examined for Golgi analysis were randomly selected from three independent experiments. Error bars indicate s.e.m. values. ***p < 0.0001. (*c*) Interphase spermatocytes from wild-type and *omt /Df* mutants expressing GFP-Cog7 were stained for GFP (Cog7), Lva and DNA. (*d*) GFP-Cog7 levels in the Golgi were quantified as mean fluorescence intensity of GFP-Cog7 in Lva-positive regions (Lva^+^) of spermatocytes from wild-type and *omt /Df* mutant males expressing GFP-Cog7. In total, we examined *N* = 140 Golgi from *omt/Df* mutant interphase spermatocytes and compared these structures with *N* = 120 Golgi from wild-type (wt) spermatocytes. The cells examined for Golgi analysis were randomly selected from three independent experiments. Error bars indicate s.e.m. Differences are not statistically significant. (*e*) Interphase spermatocytes from wild-type and *omt/Df* mutant spermatocytes expressing GFP-Cog7 were stained for GOLPH3, GFP (Cog7) and DNA. (*f*) GOLPH3 levels in the Golgi, quantified as mean fluorescence intensity of GOLPH3 in GFP-Cog7 positive regions (GFP-Cog7^+^). *N* = 165 Golgi from *omt /Df* mutant interphase spermatocytes were compared with *N* = 115 Golgi from wild-type (wt) spermatocytes. The cells examined for Golgi analysis were randomly selected from three independent experiments. Error bars indicate s.e.m. ***p < 0.0001. Scale bars, 10 µm.

**Figure 6.**
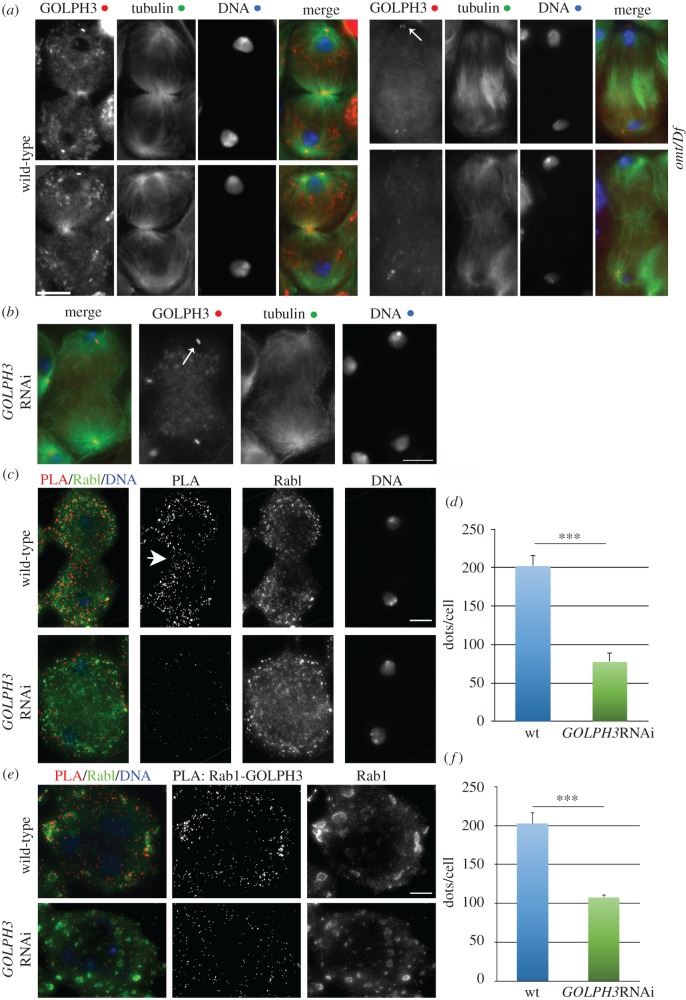
GOLPH3 protein interacts with Rab1 and requires Rab1 for localization to the cleavage furrow. (*a*) Localization of GOLPH3 protein in dividing spermatocytes. Representative images of wild-type and *omt/Df* mutant spermatocytes stained for GOLPH3, α-tubulin (Tubulin) and DNA during mid-telophase and late telophase. (*N* = 48 wild-type mid-late telophases; *N* = 44 *omt*/*Df* mutant mid-late telophase cells; the cells examined were from three independent experiments). (*b*) Knockdown of GOLPH3 protein in dividing spermatocytes from males expressing *UAS::GOLPH3RNAi* under the control of *Bam-GAL4 (GOLPH3RNAi).* Dividing spermatocytes were stained for GOLPH3, α-tubulin (tubulin) and DNA during mid-telophase. Note the defective central spindle caused by depletion of *GOLPH3.* (*c–f*) Proximity ligation assay (PLA) to visualize Rab1/GOLPH3 interaction in fixed spermatocytes. PLA with antibodies against Rab1 (mouse anti-Rab1 S12085a) and GOLPH3 (rabbit anti-GOLPH3 G49139/77) was used to test the interaction in interphase and telophase spermatocytes stained for DNA. Negative control experiments were performed with antibodies against Rab1 (mouse anti-Rab1 S12085a) and GOLPH3 (rabbit anti-GOLPH3 G49139/77) in testes from males expressing *UAS::GOLPH3RNAi* under the control of *Bam-GAL4*. Knockdown of GOLPH3 was confirmed by parallel staining for GOLPH3 and tubulin of one testis from the same individual, as shown in (*b*). Arrowhead points to PLA signals at the cleavage site. Centriole staining (white arrows) by anti-GOLPH3 is not specific [23]. Scale bars, 10 µm. (*d*,*f*) Average number (±s.e.m.) of PLA dots per cell (see Material and methods for details), in telophase (*d*) and interphase spermatocytes (*f*) from either wild-type or *GOLPH3*RNAi males. Statistically significant differences are ***p < 0.0001.

Our previous work described alterations of Golgi structure in testes of other membrane-trafficking mutants, including *GOLPH3* and *Cog7* mutants [11,17,23]. To examine the functional dependence between Rab1 and GOLPH3, *sau**^z2217^***/*Df(2L)Exel7010* (*GOLPH3*) mutant spermatocytes were fixed and stained for Rab1 and *omt*/*Df* mutants were stained for GOLPH3 (figure 5)*.* Interphase spermatocytes from *GOLPH3* displayed a concentration of Rab1 at the Golgi that was fully comparable with control (figure 5*a*,*b*). Similar immunofluorescence experiments indicated that Golgi Rab1 localization was decreased by 40% in *Cog7^z4495^/Df(3R)BSC861* (*Cog7*) mutants and that Cog7-GFP localization at the Golgi was not affected in *omt*/*Df* (figure 5*a*–*d*)*.* Conversely, *omt*/*Df* mutant spermatocytes displayed a significant reduction of Golgi-localized GOLPH3 protein (figure 5*e*,*f*)*.* Consistent with the previous findings that human Rab1A and Rab1B are essential to recruit the ArfGEF GBF1 at the Golgi [30,52], mutations in *Rab1* disrupted Golgi localization of the GBF1 orthologue Garz [53] in interphase spermatocytes (electronic supplementary material, figure S5*a*). Mutations in *Rab1* also impaired localization of GOLPH3 protein to the cleavage furrow of dividing spermatocytes. All telophase spermatocytes from wild-type displayed accumulation of GOLPH3 protein at the poles and at the cleavage site (100% of mid-telophases, *N* = 48). In contrast with wild-type, 84% of mid-telophases from *omt*/*Df* mutant males failed to accumulate GOLPH3 to both the poles and the cleavage site (*N* = 44; figure 6*a*). We next checked whether *omt/Df* mutant testes express reduced levels of GOLPH3 compared with wild-type testes. This analysis failed to reveal a significant reduction of GOLPH3 expression level in *omt/Df* mutant testes (figure 3*a*,*b*). Taken together these results indicate that mutations in *Rab1* cause mislocalization of GOLPH3 in both interphase and dividing spermatocytes.

### 2.6. Rab1 interacts with Garz and GOLPH3

**Figure 7.**
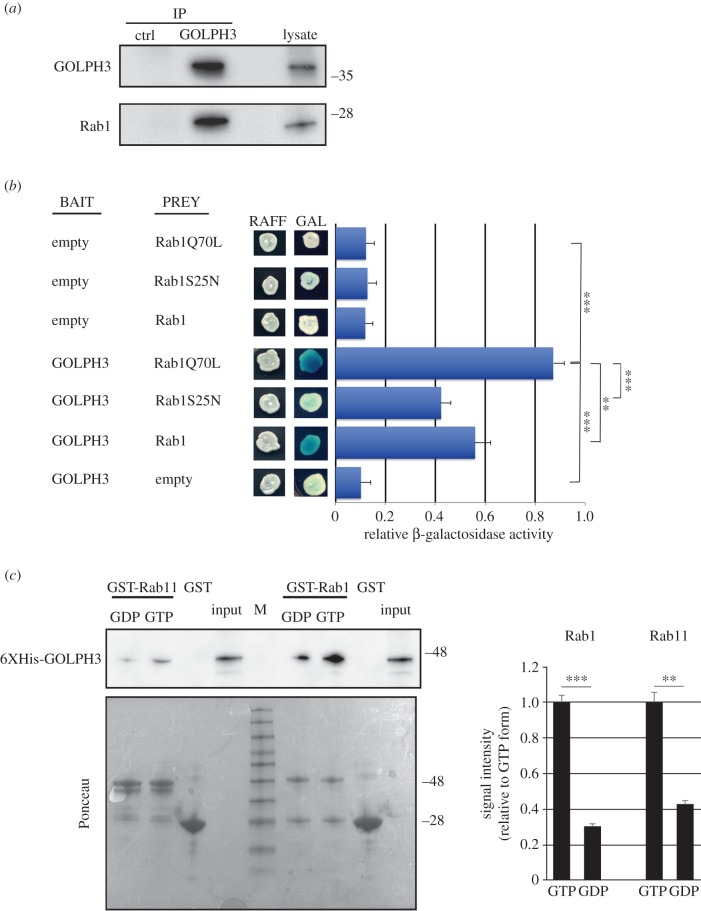
GOLPH3 protein interacts with Rab1. (*a*) Protein extracts from wild-type testes were immunoprecipitated with antibodies against *Drosophila* GOLPH3 (rabbit G49139/77) and blotted with mouse anti-GOLPH3 S11047/1/56 or with mouse anti-Rab1 S12085a antibodies. Preimmune serum (G49139/1, from the same animal before the immunization) was used as control (ctrl). Two per cent of the total lysate and 1/3 of the immunoprecipitates were loaded and probed with the indicated antibody. Molecular masses are in kilodaltons. The Co-IP experiment was performed three times with identical results. (*b*) Yeast two-hybrid assay: yeast cells cotrasformed with *GOLPH3* bait plasmid together with a prey plasmid containing the indicated coding sequence were grown in X-gal-containing plates. In the presence of the *GOLPH3* bait, Rab1Q70L and wild-type Rab1 proteins induce LacZ expression (blue colour indicates positive interaction). Quantification of LacZ reporter expression (graph) induced with different combinations of bait and prey plasmids is shown. Error bars indicate s.e.m. Statistically significant differences are **p < 0.01 (*p* = 0.0071), ***p < 0.0001. RAFF, raffinose; GAL, galactose. (*c*) Recombinant GST-Rab11 and GST-Rab1 proteins, immobilized on glutathione beads and loaded with either GDP-β-S (GDP) or GMP-PNP (GTP) were incubated with recombinant 6XHis-tagged GOLPH3. The amount of 6XHis-tagged GOLPH3 that directly bound to each GST-Rab protein or to GST was detected with anti-6XHis antibodies. Ponceau staining is shown as a loading control. 0.1% of the input and 25% of the pull-down were loaded and probed with the indicated antibody. Molecular masses are in kilodaltons. M, molecular mass marker. Graph represents quantification of 6XHis-tagged GOLPH3 binding to each form of Rab by western blot. Protein band intensities were obtained from three independent experiments. Statistically significant differences are **p < 0.01, ***p < 0.0001.

**Figure 8.**
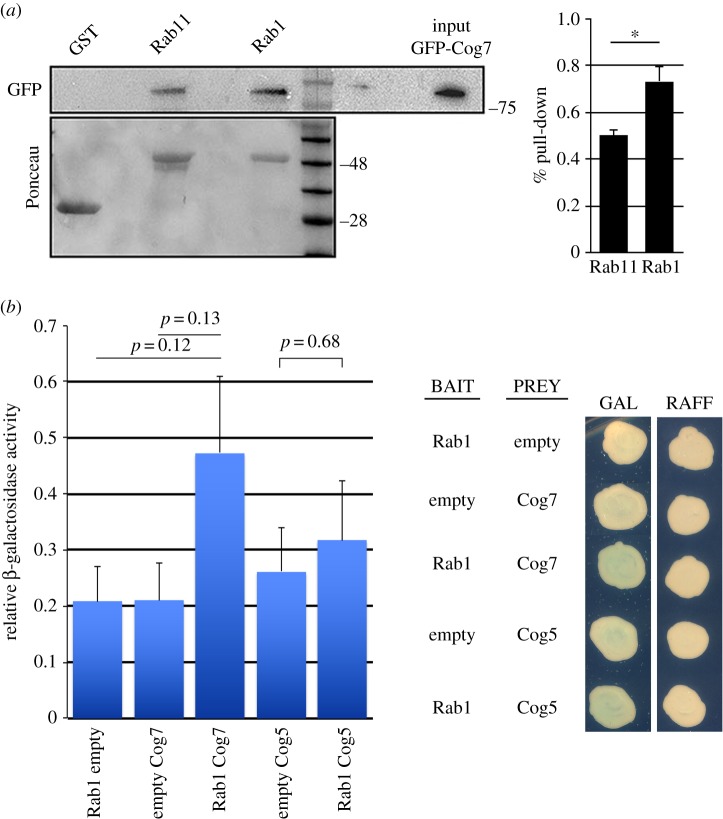
GST pull-down and yeast two-hybrid assay were used to test the interaction of *Drosophila* Cog5 and Cog7 with Rab1. (*a*) Bacterially expressed GST-Rab1, GST-Rab11 and GST were purified by Gluthatione-Sepharose beads and incubated with larval brain lysates from animals expressing GFP-Cog7. GST-Rab11 (Rab11) and GST-Rab1 (Rab1), but not GST, precipitated GFP-Cog7 from brain protein extracts. Note that a more robust interaction is obtained with GST-Rab1. Ponceau staining is shown as a loading control. Two per cent of the input and 25% of the pull-downs were loaded and probed with the indicated antibody. Molecular masses are in kilodaltons. The graph represents quantification of the amount of GFP-Cog7 that was pulled down from each form of Rab in western blotting analysis. The protein band intensities were obtained from three independent experiments. *p < 0.05. (*b*) Yeast two-hybrid assay: yeast cells cotrasformed with Rab1 bait plasmid together with a prey plasmid containing the indicated coding sequence were grown in X-gal-containing plates. Quantification of LacZ reporter expression (graph) induced with different combinations of bait and prey plasmids. Error bars indicate s.e.m. values. The differences are not statistically significant.

Our findings indicate that Rab1 colocalizes with GOLPH3 and Cog7 at the Golgi and raise the question whether these proteins could physically interact. To test Rab1 and GOLPH3 interaction, we used co-immunoprecipitation (Co-IP) assay. Rab1 coimmunoprecipitated with GOLPH3 in testis extracts (figure 7*a*). In agreement with previous work in mammalian cells [30], our Co-IP experiments also revealed the interaction of Rab1 with the GBF1 protein Garz (electronic supplementary material, figure S5*b*). To determine if the interaction between Rab1 and GOLPH3 was dependent on the GTP-binding state of Rab1 we used yeast two-hybrid assays to assess the interaction of GOLPH3 with wild-type Rab1, Rab1Q70L (constitutively active mutant) and Rab1S25N (dominant-negative mutant). Wild-type GOLPH3 exhibited stronger binding interaction with Rab1Q70L and the weakest binding with Rab1S25N, suggesting that GOLPH3 might be a Rab1 effector (figure 7*b*). Finally, using purified recombinant proteins, the interaction between GOLPH3 and Rab1 was demonstrated to be direct and to be dependent on Rab1 binding to GTP (figure 7*c*,*d*). We next tested the interaction between Rab1 and the COG complex by using glutathione *S*-transferase (GST) pull-down analysis and yeast two-hybrid assays (figure 8*a*,*b*). Although GFP tagged Cog7 was pulled down by both GST-Rab11 and GST-Rab1 (but not GST), from larval brain lysates, this experiment indicated a more robust interaction with GST-Rab1 (figure 8*a*). However, consistent with previous data in mammalian cells [54], our yeast two-hybrid analysis failed to indicate a direct interaction of Rab1 with either Cog7 or Cog5 proteins (figure 8*b*). Taken together these results suggest that Rab1 interaction with the COG complex must be mediated by COG subunits other than Cog5 and Cog7. To further investigate Rab1/GOLPH3 interaction *in situ* in fixed cells, we used a proximity ligation assay (PLA) assay to map sites where Rab1 and GOLPH3 are in close proximity (figure 6*b–f*). PLA assay confirmed the interaction of GOLPH3 with Rab1 in fixed interphase and telophase spermatocytes. Remarkably PLA signals were found in close proximity with Golgi stacks and Golgi derived vesicles, suggesting the requirement for GOLPH3/Rab1 interaction for secretory vesicle trafficking (figure 6*c–f*). In addition, PLA signals were found enriched in the cleavage site of wild-type dividing spermatocytes, suggesting that Rab1 and GOLPH3 co-function during furrow ingression (figure 6*c*).

## 3. Discussion

The evolutionarily conserved small Rab1 GTPase is known to control ER-to-Golgi and intra-Golgi vesicle trafficking [25,26]. Here we have provided the first comprehensive demonstration for Rab1 function in cytokinesis, in tissues of a multicellular organism. A possible involvement of Rab1 in mitotic cytokinesis was previously suggested by a genome-wide screen aimed at identifying genes required for cytokinesis in cultured *Drosophila* cells, reporting a slight increase of binucleate cells in RNAi-treated cells when compared with control [55]. Our analysis reveals defects in early stages of cytokinesis of dividing spermatocytes, neuroblasts and S2 cells with reduced Rab1 protein expression, which result in incomplete contractile ring constriction. Although myosin II/anillin rings were observed in early telophases of *omt*/*Df* mutants, these structures failed to undergo full constriction during cytokinesis. A similar phenotype was found also in *Drosophila* S2 cells depleted of Rab1. Failure to assemble functional actomyosin rings is a commonly observed phenotype in male meiotic mutants of membrane-trafficking components including the COG subunits Cog5 and Cog7 [10,11], the Arf6 and Rab11GTPases [14,15], the TRAPPII subunit Brunelleschi [13] and GOLPH3 [23]. A model has been suggested whereby, during cytokinesis, assembly and dynamics of the contractile apparatus are intimately connected with vesicle trafficking and membrane remodelling at the cleavage furrow [24]. In this context, membrane vesicles that fuse with the furrow membrane during cytokinesis might also transport structural components of the contractile ring or F-actin regulators. Indeed, visualization of actin and endocytic vesicles in cellularizing *Drosophila* embryos has suggested that F-actin and vesicles might be targeted as a unit to the furrow site [56]. Our finding that Rab1 localizes at the Golgi suggests a role for this protein in Golgi trafficking during cytokinesis. In agreement with this hypothesis, our analysis of Golgi in interphase spermatocytes by 3D-SIM super-resolution microscopy and TEM revealed a highly altered structure in *Rab1* mutants, suggesting that defective trafficking through the Golgi may impair the flow of vesicle trafficking to the cleavage site and halt cytokinesis. Moreover, the characteristic organization of *Drosophila* Golgi into multiple discrete stacks [57], scattered throughout the cytoplasm, allowed us to uncover structural defects, caused by *Rab1* mutations, that might not be identified in mammalian cells where the stacks are interconnected into a single ribbon-like Golgi. Indeed, mutations in *Rab1* affected both the number and size of the Golgi stacks and disrupted the ultrastructure of Golgi cisternae. Golgi fragmentation is likely to result from defective COP I-mediated vesicle trafficking, which in turn depends on the GTPase Arf1 and its guanine nucleotide exchange factor GBF1 [58]. Consistent with this hypothesis, our work demonstrates that Rab1 interacts with Garz, the *Drosophila* orthologue of GBF1, which is essential to recruit this protein to the Golgi. Golgi fragmentation and trafficking defects are also likely to result from decreased localization of the PI(4)P-binding protein GOLPH3. Remarkably, GOLPH3 is a key protein for maintaining Golgi architecture and vesicular release [59]. A recent study has proposed that human Rab1B, in complex with PITPNC1, might control Golgi morphology by regulating Golgi PI(4)P levels and hence indirectly the abundance of the PI(4)P effector GOLPH3 [44]. In agreement with this work, our data indicate that GOLPH3 requires wild-type function of Rab1 for its localization at the Golgi membranes during both interphase and telophase. Moreover, we provide evidence that GOLPH3 protein directly interacts with Rab1-GTP and requires wild-type function of Rab1 for its recruitment to the cleavage site. Taken together our data suggest that Rab1 protein, by contributing to GOLPH3 recruitment, enables secretory vesicle trafficking and actomyosin constriction during cytokinesis. We cannot exclude that the loss of Rab1 could have additional effects through other Golgi effectors in addition to GOLPH3 and that the cytokinesis defects might be the indirect consequences of altered secretory or endocytic pathways that are known to be important for cytokinesis. Indeed mutations in *Rab1* do not affect Golgi localization of Cog7 but disrupt recruitment of the ArfGEF orthologue Garz, a known Golgi effector of Rab1. Nevertheless, our data indicate GOLPH3 is a major effector of Rab1 in mediating contractile ring constriction and cleavage furrow ingression during cytokinesis.

In mammalian cells, a single molecular TRAPPII complex acts as a GDP–GTP exchange factor for Rab1 [60]. This complex appears to have a counterpart in *Drosophila melanogaster* where Bru, the fly orthologue of the TRAPPII subunit Trs120p, is also required for cleavage furrow ingression during male meiotic cytokinesis [13,61]. In fission yeast and plant cells, this role appears to require both the TRAPPII and exocyst complexes that associate with vesicles in the cleavage furrows [62,63]. These data suggest a possible conserved role for Rab1 together with the TRAPPII complex in guiding membrane addition to the cleavage furrow during cytokinesis that in animal cells may be played by a single complex. The investigation of such possibilities will be the topic of future work.

## 4. Material and methods

### 4.1. Fly stocks and transgenes

Flies were raised at 25°C by standard procedures. Oregon-R flies were used as wild-type controls. *omt^z4144^*, *Cog7^z4495^* and *sau^z2217^* mutant strains were described previously [11,23,45] and were from the C. Zuker collection [45]. *UAS::GOLPH3-RNAi* flies were described previously [23] and were obtained from the Vienna *Drosophila* RNAi Collection (VDRC line 46150) and driven in spermatocytes using *Bam-GAL4* [64]. The chromosomal deficiencies *Df(3R)ED10838*, *Df(3R)GC14*, *Df(3R)ED10845*, *Df(3R)BSC861*, *Df(2L)Exel7010* and the P elements *Rab1^S147213^* and *Rab1^e01287^* were obtained from the Bloomington Drosophila Stock Center (Indiana University, Bloomington, IN, USA). Flies expressing GFP-Cog7 were described previously [11].

### 4.2. Molecular biology and rescue experiments

DNA sequencing of Rab1 protein coding exons was performed from individuals carrying the *omt^z4144^* mutation and the original Zuker-background chromosome as described previously [13]. To generate the *YFP-Rab1* fusion construct, the CDS of YFP was fused in frame to the N-terminus of the *Rab1* cDNA. *YFP-Rab1* was cloned into the pCaSpeR-sa that contains the spermatocyte specific *sa* promoter and SV40 terminator (see Szafer-Glusman *et al.* [47] for details on this vector). To generate the RFP-Rab1 fusion construct, the cDNA of *Rab1* was cloned into pCasper4-tubulin [23] in frame with N-terminal mRFP sequence. Transgenic flies were generated by P-element mediated germline transformation, performed by Bestgene Inc. (Chino Hills, CA, USA). *YFP-Rab1* and *RFP-Rab1* were crossed into the *omt/Df* mutant background to test for phenotypic rescue of male sterility and meiotic cytokinesis failure. *RFP-Rab1*, crossed into the *omt/Df* mutant background, was used to test for phenotypic rescue of lethality. To obtain GST fusion Rab1/Rab11 proteins, full-length cDNAs corresponding to *Drosophila Rab1* and *Rab11* were cloned into a pGEX-6p-2 (GE Healthcare), as described previously [23]. Male fertility tests were performed in at least 10 vials by crossing five males of either Oregon-R or mutant genotypes to 5 Oregon-R virgin females and counting the number of larvae in each vial.

### 4.3. Protein expression and purification, antibody generation

GST-full-length *Drosophila* Rab1 protein was expressed in BL21-CodonPlus (DE3) cells (Invitrogen) and purified using HiTrap affinity columns (GTtrap FF, and GSTtrap HP columns, GE Healthcare) operated with AKTA 900 fast protein liquid chromatography. Polyclonal antisera against the purified GST-Rab1 protein were produced in rabbits and mice (Agro-Bio Services; www.agro-bio.com). The anti-GST-Rab1 antisera were first depleted of anti-GST antibodies and then affinity-purified against GST-Rab1. The pET Directional TOPO Expression kit (Thermofisher Scientific) was used to obtain 6XHis-GOLPH3. 6XHis-GOLPH3 was purified using cOmplete His-Tag Purification Resin (Roche). The following antibodies were used in the assays of this study: mouse anti-Rab1 antibody S12085a and rabbit anti-Rab1 L12085a/169 antibody.

### 4.4. Western blotting and co-immunoprecipitation

Immunoblotting analysis of Rab1 protein was performed from protein extracts of either adult testes or larval brains. Briefly, 30 adult testes or 20 brains from males of each genotype were homogenized on ice in 100 µl Lysis buffer (10 mM Tris–HCl pH 7.5, 150 mM NaCl, 0.5 mM EDTA, 0.5% NP40, 1 mM PMSF, 1× protease inhibitor cocktail) using a Dounce homogenizer. Samples were separated on Mini-protean TGX precast gels (Bio-Rad) and blotted to PVDF membranes (Bio-Rad). Membranes were blocked in Tris-buffered saline (Sigma-Aldrich) with 0.05% Tween-20 (TBST) containing 5% non-fat dry milk (Bio-Rad; Blotting Grade Blocker) for 3–4 h at room temperature followed by incubation with primary and secondary antibodies diluted in TBST. Co-IP experiments from testes expressing RFP-tagged proteins were performed using the RFP trap-A kits purchased from ChromoTek (Planegg-Martinsried), following the protocol that was previously described [23]. To immunoprecipitate *Drosophila* GOLPH3, we used the protocol described previously [23]; 400 adult testes were homogenized in 500 µl of Lysis buffer (see above) for 40 min on ice. The testis lysate was precleared and divided into two. Fractions were incubated with either 5 µg of rabbit anti-GOLPH3 G49139/77 antibody or 5 µg of rabbit pre-immune serum (G49139/1, from the same animal before the immunization). After antibody incubation, immunoprecipitation was performed using the immunoprecipitation kit-Protein G (Roche) following the manufacturer's instructions. Primary antibodies, used for immunoblotting were as follows: mouse anti-GOLPH3 S11047/1/56 (1 : 2500; [23]) and rabbit anti-GOLPH3 (1 : 2500; G49139/77 [23]), mouse monoclonal anti-α-tubulin (1 : 500; Sigma-Aldrich T6199), mouse anti-Rab1 antibody S12085a (1 : 750; this study), rabbit anti-Rab1 L12085a/169 (1 : 1000; this study**)**, rabbit anti-Garz ([53], gift of Dr A. Paululat, University of Osnabruck, Germany, 1 : 500), mouse monoclonal anti-RFP (1 : 1000; Chromotek, 6G6), mouse HRP anti-GFP (1 : 1000; Vector-Lab) and mouse anti-6XHis (1 : 1000, Invitrogen). HRP-conjugated secondary antibodies (GE Healthcare) were used at 1 : 5000. After incubation with the antibodies, blots were washed in TBST and imaged using an ECL detection kit (GE Healthcare).

### 4.5. GST pull-down assays

GST, GST-Rab1 and GST-Rab11 proteins were expressed in bacteria and purified using Glutathione-Sepharose 4B beads (GE Healthcare) following the manufacturer's instructions. At least 200 larval brains were homogenized for 40 min on ice in 500 µl of Lysis buffer (25 mM Tris–HCl pH 7.4, 150 mM NaCl, 0.5% NP-40, 1 mM EDTA) with the addition of protease and phosphatase inhibitor cocktails (Roche), using a Dounce homogenizer. After clearing the lysates by centrifugation, protein concentration of the supernatants was determined by Bradford assay (Bio-Rad). GST pull-down was performed by incubating larval brain lysates with either GST, GST-Rab1 or GST-Rab11 (at the appropriate concentration) bound to Glutathione-Sepharose 4B beads (GE Healthcare), with gentle rotation, at 4°C for 2 h. After rinsing in ‘wash buffer’ (25 mM Tris–HCl pH 7.4, 150 mM NaCl, 1% NP-40, 1 mM EDTA, protease and phosphatase inhibitors), for three times, the beads were boiled in SDS sample buffer, and separated by SDS-PAGE. The bound proteins were analysed by western blotting (see above). Before immunoblotting, PVDF membranes were stained with Ponceau (Sigma-Aldrich). Direct interaction between 6XHis-GOLPH3 and either GST-Rab1 or GST-Rab11 proteins was carried out using the protocol described previously [4]. 6XHis-GOLPH3 was incubated with GST or GST-Rab proteins for 5 h at 4°C. 6XHis-GOLPH3 bound to ether GST, GST-Rab1 or GST-Rab11 was detected by western blotting analysis using mouse anti-6XHis antibodies (1 : 1000). Guanosine 5′-[β-thio]diphosphate trilithium salt (GDP-β-S) and guanosine 5′-[β,γ-imido]triphosphate trisodium salt hydrate (GMP-PNP) were purchased from Sigma-Aldrich.

### 4.6. Immunofluorescence staining and microscopy

Cytological preparations were made with brains and testes from third instar larvae or S2 cells. To visualize Cog7-GFP or RFP-Rab1, larval testes were fixed in 4% methanol-free formaldehyde (Polysciences, Warrington, PA, USA), as previously described [9–11]. Following fixation, testes were incubated with either mouse anti-GFP (3E6, Thermofisher Scientific), or rat anti-RFP (ChromoTek), diluted 1 : 200 in phosphate buffered saline (PBS). To visualize α-tubulin with either Rab1 or GOLPH3, testes were fixed with methanol and formaldehyde according to Frappaolo *et al.* [9]. For immunostaining of larval testes and brains with other antibodies, preparations were fixed using 3.7% formaldehyde in PBS and then squashed in 60% acetic acid as previously described [9,65]. S2 cells used in immunostaining experiments were harvested 72 h after transfection for RNAi, and plated on glass coverslips for 1 h. S2 cells were then fixed with 4% paraformaldehyde in PHEM buffer (60 mM PEPES, 25 mM HEPES, 10 mM EGTA, 4 mM MgCl_2_). Cells were then permeabilized in PBS with 1% Triton X-100 and 3% BSA and washed three times with PBSTB (PBS 1× with 0.1% Triton X-100 and 1% BSA). Monoclonal antibodies were used to stain α-tubulin (1 : 300; Sigma-Aldrich, T6199), rabbit anti-lamin (dilution 1 : 50, [66]) and anti-GFP (see above). Polyclonal antibodies were as follows: rabbit anti-Lva (1 : 500; [50]), gift from O. Papoulas (University of Texas at Austin); anti-GOLPH3 G49139/77 [23], diluted 1 : 1000; mouse anti-Rab1 antibody S12085a (1 : 750; this study), rabbit anti-Rab1 L12085a/169 (1 : 1000; this study), rabbit anti-Pav (1 : 750; [49]), rabbit anti-Garz (1 : 250; [53]), gift from A. Paululat; rabbit and mouse anti-anillin (1 : 1000; [17]), rabbit anti-myosin II (1 : 400; [48]) gift of R. Karess, Paris Diderot University. F-actin in S2 cells was visualized with Alexa Fluor 594 phalloidin (dilution 1 : 50, Invitrogen). Secondary antibodies were: Alexa 555-conjugated anti-rabbit IgG (1 : 300, Life Technologies), and FITC-conjugated anti-mouse/anti-rat IgG (1 : 30, Jackson ImmunoResearch). All incubations with primary antibodies (diluted in PBT containing 3% BSA) were performed overnight at 4°C. Incubations with secondary antibodies were performed at room temperature for 1 h. After immunostaining, samples were rinsed in PBS and mounted in Vectashield mounting medium with DAPI (H-1200, Vector Laboratories). Images were captured with a charged-coupled device (CCD camera, Qimaging QICAM Mono Fast 1394 Cooled), connected to a Nikon Axioplan epifluorescence microscope equipped with an HBO 100-W mercury lamp and 40× and 100×objectives. Spermatocyte Golgi stacks were imaged at 0.5 µm steps through the whole cell using an Olympus FV1000 confocal microscope with a 60× 1.4 NA lens. Golgi stack number and size were measured using the Analyse Particles tools in ImageJ/Fiji. Super-resolution images (figure 4) were captured at 21°C on a DeltaVision OMX V3 Blaze microscope (GE Healthcare, UK) equipped with a 60×/1.42 oil UPlanSApo objective (Olympus). The raw data were computationally reconstructed with SoftWorx 6.1 (GE Healthcare) using a Wiener filter setting of 0.006. Images from spermatocytes treated for PLA assays were captured with a charged-coupled device (Axiocam 503 mono CCD camera), and the ZEN2 software, connected to a Zeiss Cell Observer Z1 microscope equipped with an HXP 120 V inclusive built in power supply, lamp module and a 63×/1.4 objective. Images were analysed with ImageJ and processed in Photoshop. The protein content of Golph3, Cog7-GFP or Rab1 in the Golgi membranes (figure 5) was measured using ImageJ software. In detail, the Golgi compartment was demarcated using the freehand selection tool and mean signal intensity of the protein was measured in the selected compartment.

### 4.7. Proximity ligation assay

PLA was performed as described previously [9]. Preparations fixed using 3.7% formaldehyde in PBS and then squashed in 60% acetic acid were blocked by immersing in the blocking solution contained in the kit (Duolink In Situ PLA Probes, Sigma-Aldrich), following the instructions provided by the manufacturer. After blocking, samples were incubated with primary antibodies (rabbit anti-GOLPH3 G49139/77, diluted 1 : 1000; and mouse anti-Rab1 S12085a, 1 : 750) diluted in the Duolink *In Situ* Antibody Diluent included in the kit (Duolink *In Situ* PLA Probes, Sigma-Aldrich) in a humid chamber overnight at 4°C. The PLA probe incubation and the detection protocol were performed in accordance with the procedures described in the Duolink *In Situ*-Fluorescence User Guide, using the Duolink *In Situ* PLA Probes and Duolink *In Situ* Detection Reagents. Following the detection steps, specimens were mounted in Vectashield Mounting Medium containing DAPI. Quantification of the number of PLA signals per cell was obtained using the Analyse Particles tools of the ImageJ software. In detail, per each genotype, 20 cells were randomly selected in the images collected from three experiments and manually demarcated using the freehand selection tool. The ‘analyse particles’ command in ImageJ was then used to count the number of dots in the selected area.

### 4.8. Yeast two-hybrid assay

The assay was performed using the B42/lexA system with strain EGY48 (*Mata his3 ura3 trp1 6lexAOP-LEU2*; *lexAOP-lacZ* reporter on plasmid pSH18-34) as the host strain [67]. This strain was cotransformed with various combinations of bait (pEG202) and prey (pJG4-5) plasmids carrying *GOLPH3* and *Rab1* cDNAs or *Rab1*, *Rab1S25N*, *Rab1Q70 L*, *Cog5* and *Cog7* cDNAs. To assess two hybrid interaction, the strains were spotted on 5-bromo-4-chloro-3-indolyl-β-d-galactopyranoside (X-GAL) selective synthetic plates containing either raffinose (RAFF, prey not induced) or 2% galactose (GAL, prey expressed) [68]. To quantify the yeast two-hybrid results, a β-galactosidase assay was performed. Strains were first grown in a selective medium containing galactose, then cells were collected and protein extracts were prepared in order to test β-galactosidase enzyme activity using *o*-nitrophenyl-d-galactoside (ONPG) as a substrate, as described previously [21]. For each sample, five independent transformants were used and the assays were performed in duplicate to calculate average β-galactosidase units and standard error.

### 4.9. Transmission electron microscopy

Male mid-pupae were dissected in PBS under a stereo light microscope. Testes were isolated and fixed in 2.5% glutaraldehyde buffered in PBS overnight at 4°C. Samples were post-fixed with 1% osmium tetroxide in PBS for 1 h at 4°C. After careful rinsing, the material was dehydrated through a graded series of ethanol, embedded with a mixture of Epon-Araldite resin and polymerized at 60°C for 48 h. Ultrathin sections (65–75 nm) were cut with a Reichert ultramicrotome equipped with a diamond knife, collected with formvar-coated copper grids, and routinely stained with uranyl acetate and lead citrate. TEM preparations were observed with a FEI Tecnai G2 Spirit transmission electron microscope operating at an accelerating voltage of 100 kV and equipped with a Morada CCD camera (Olympus).

### 4.10. *Drosophila* cell culture and RNAi-mediated interference

The DMel strain of *Drosophila* S2 cells (Invitrogen) was used in all experiments. These cells were cultured in serum-free medium (Express Five; GIBCO) supplemented with 1 mM glutamine, penicillin and streptomycin at 25°C. Generation of all dsRNAs was performed as previously described [69]. Oligonucleotides used in this work are as follows:

Rab1.f1TAATACGACTCACTATAGGGAGAGTTATATCAGCACAATCGGAGTGGA

Rab1.r1TAATACGACTCACTATAGGGAGATCAGCAGCAACCGGATTTGGTGTTT

Rab1BKN22849for TAATACGACTCACTATAGGGAGATTCCGGATTCACAGATGACA

Rab1BKN22849rev TAATACGACTCACTATAGGGAGAGTGTAGCCCTGGTTGGAAGA

For RNAi, cells were seeded at 1 × 10^6^ cells in a six-well plate the day before transfection. The day after, we used 20 µg of dsRNA with 20 µl of TRANSFAST (Promega) and 1 ml of medium mixed together for 15 min. The mix was then overlaid on the cells for 1 h at 25°C. After this treatment, 3 ml of complete Express 5 medium was added to the cells. RNA interference was performed for 72 h.

### 4.11. Statistical analysis

For all the immunofluorescence, differences between wild-type and mutant cells were examined for statistical significance using unpaired Student's *t*-test with Prism v. 6 (GraphPad). In western blotting analysis, the band intensities were quantified using Image Lab software (v. 4.0.1; Bio-Rad Laboratories, Hercules, CA, USA). The representative results from at least three independent experiments were analysed using unpaired Student's *t*-test with Prism v. 6 (GraphPad). For yeast two-hybrid experiments, differences between each group were examined for statistical significance using unpaired Student's *t*-test using Prism 6 (GraphPad). Data are expressed as mean ± s.e.m.

## Supplementary Material

Figure S1. Figure S1. The gene omelette encodes the Drosophila orthologue of Rab1

## Supplementary Material

Figure S2 Localization of Pav protein at the cleavage site depends on Rab1 function

## Supplementary Material

Figure S3 Validation of mouse anti-Rab1 and rabbit anti Rab1 antibodies by Western Blot and immunofluorescence assays.

## Supplementary Material

Figure S4. Localization of YFP-Rab1 protein in live dividing spermatocytes.

## Supplementary Material

Figure S5. Rab1 is required to recruit Garz to the Golgi membranes.

## Data accessibility

The datasets supporting this article have been uploaded as part of the electronic supplementary material.
