# Supplementary figures and images for: Rab1 interacts with GOLPH3 and controls Golgi structure and contractile ring constriction during cytokinesis in Drosophila melanogaster
Source: Open Biol. 2017 Jan 18;7(1):160257. doi: 10.1098/rsob.160257 (PMC5303273; doi:10.1098/rsob.160257)

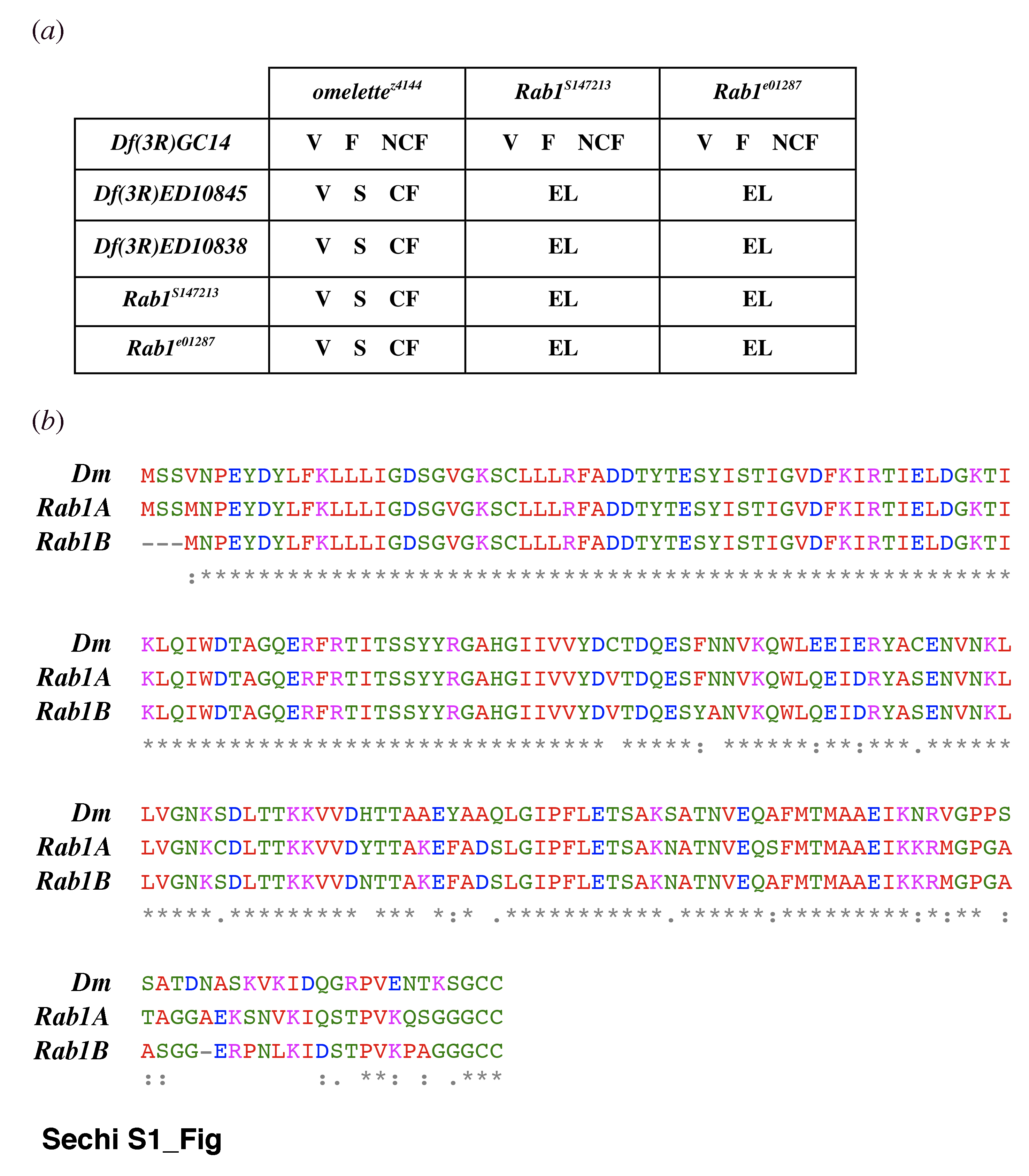

Supplement: Figure S1. Figure S1. The gene omelette encodes the Drosophila orthologue of Rab1 [file rsob160257supp1.tif]

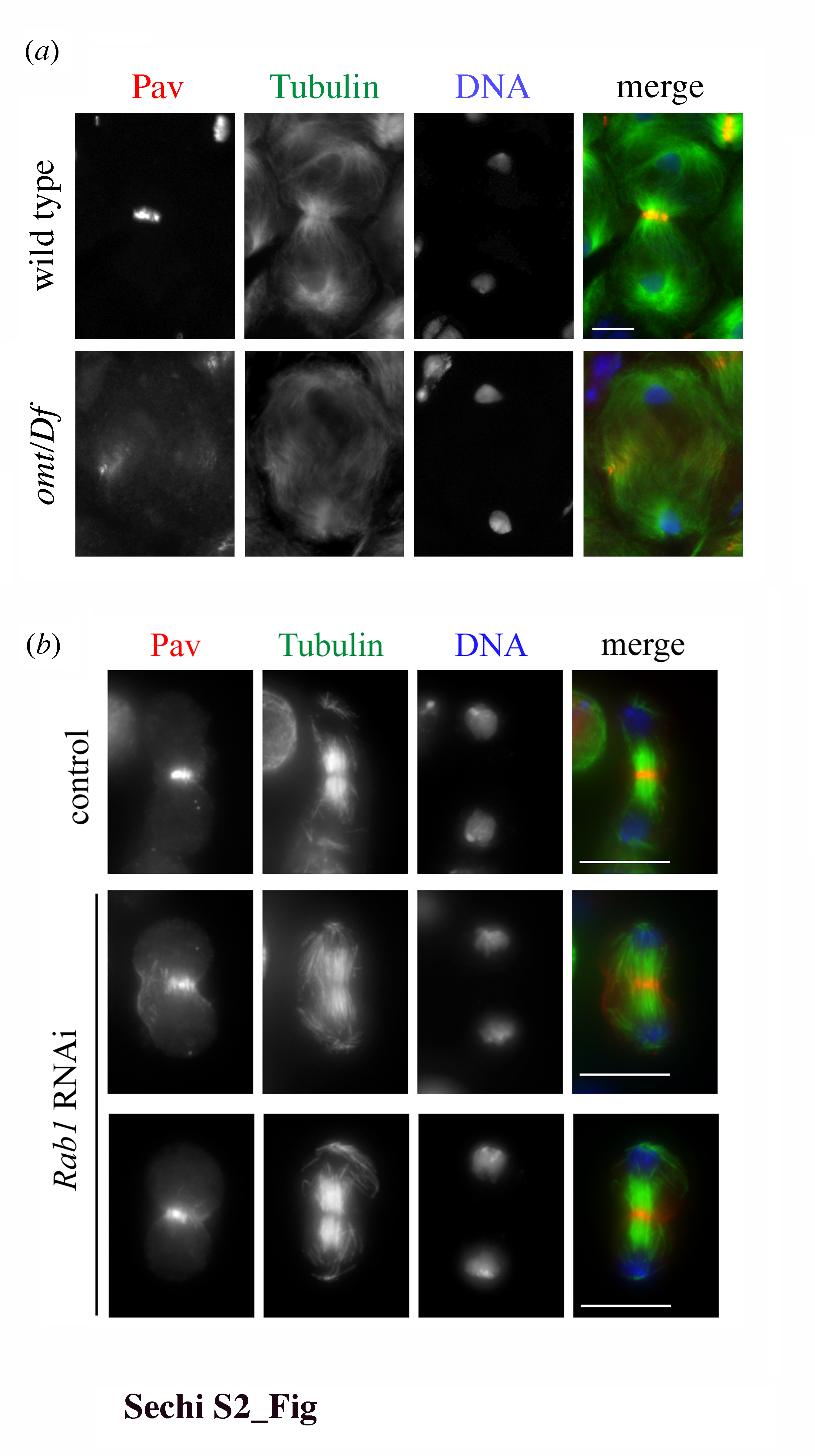

Supplement: Figure S2 Localization of Pav protein at the cleavage site depends on Rab1 function [file rsob160257supp2.tif]

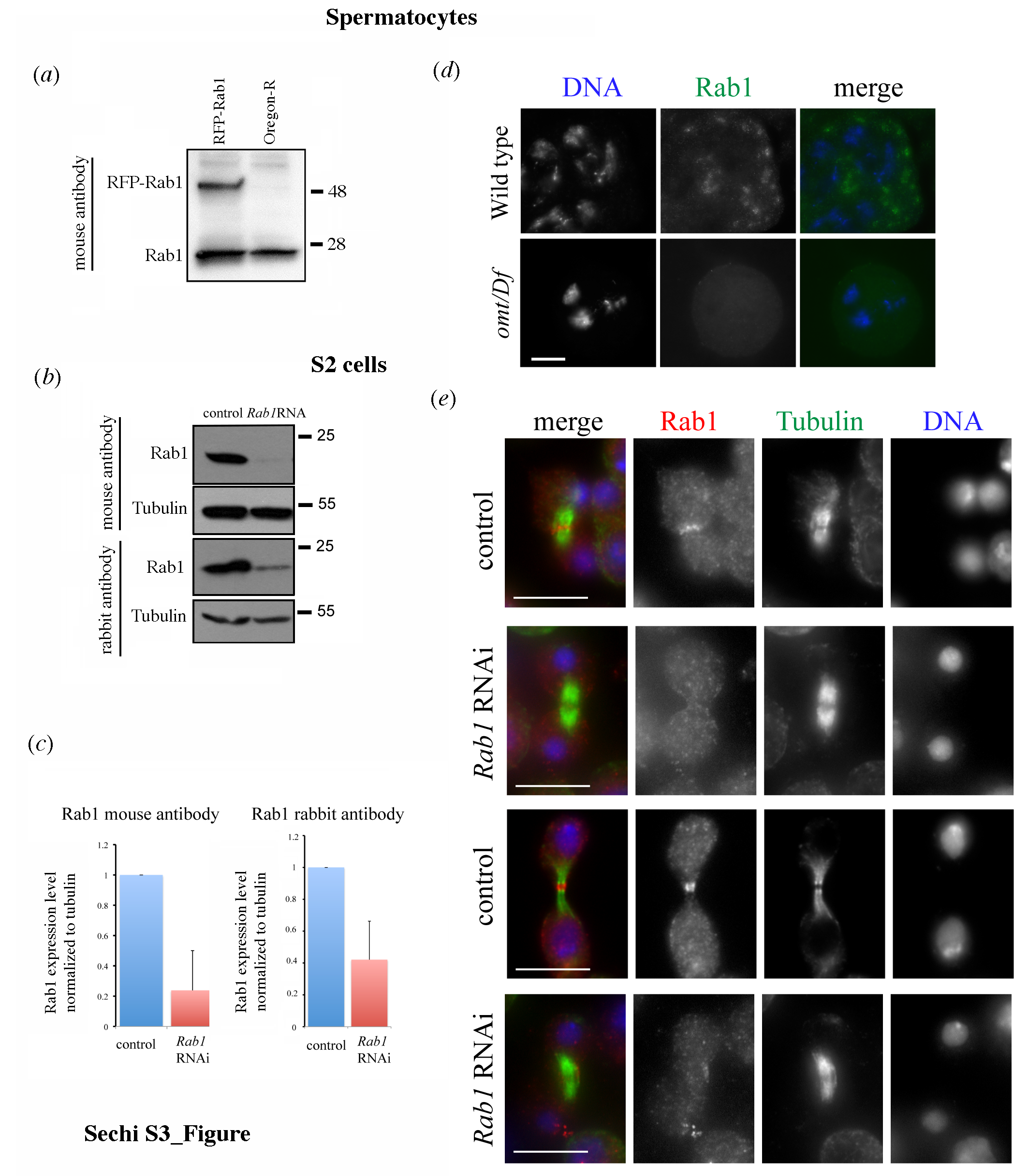

Supplement: Figure S3 Validation of mouse anti-Rab1 and rabbit anti Rab1 antibodies by Western Blot and immunofluorescence assays. [file rsob160257supp3.tif]

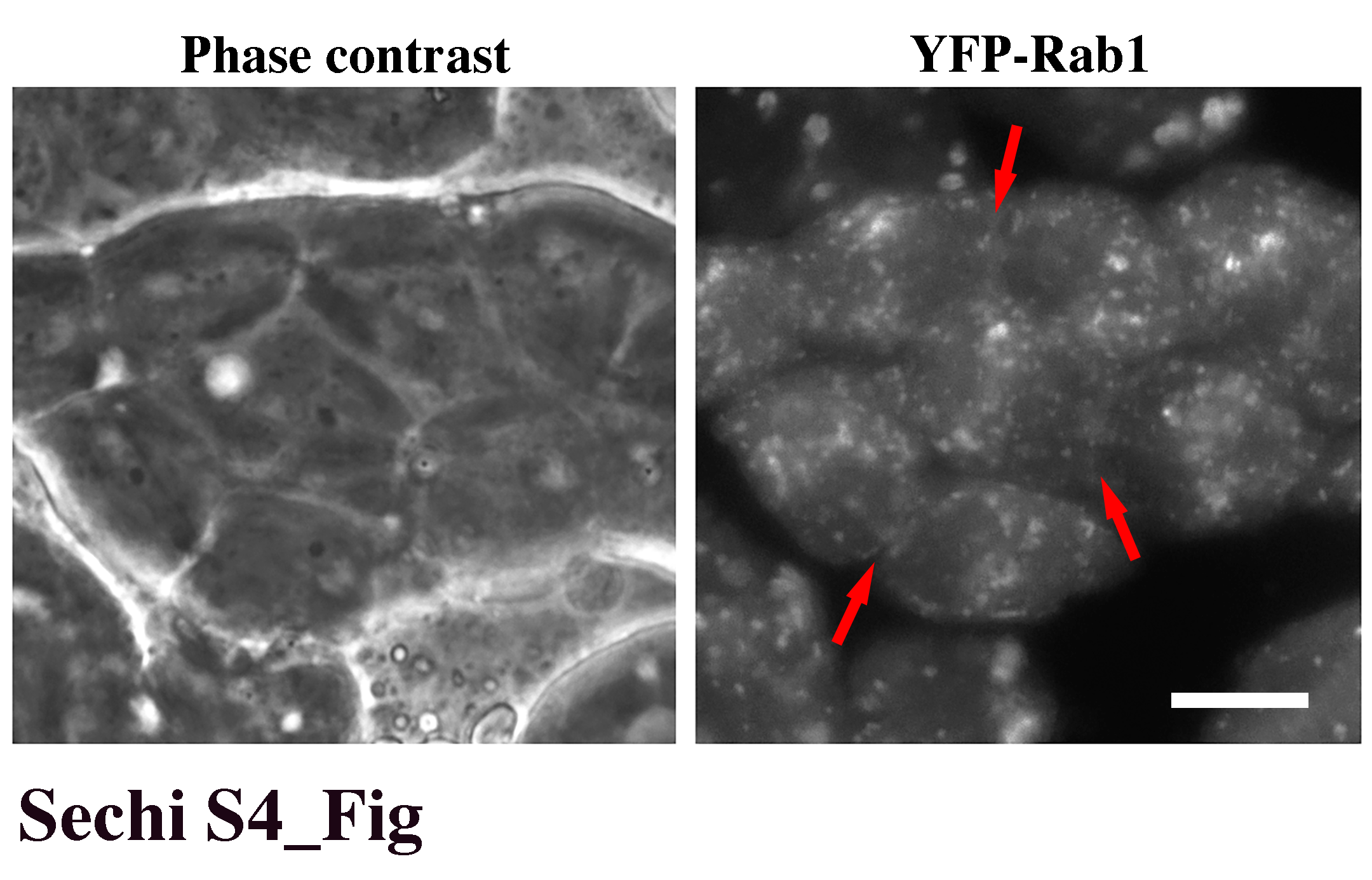

Supplement: Figure S4. Localization of YFP-Rab1 protein in live dividing spermatocytes. [file rsob160257supp4.tif]

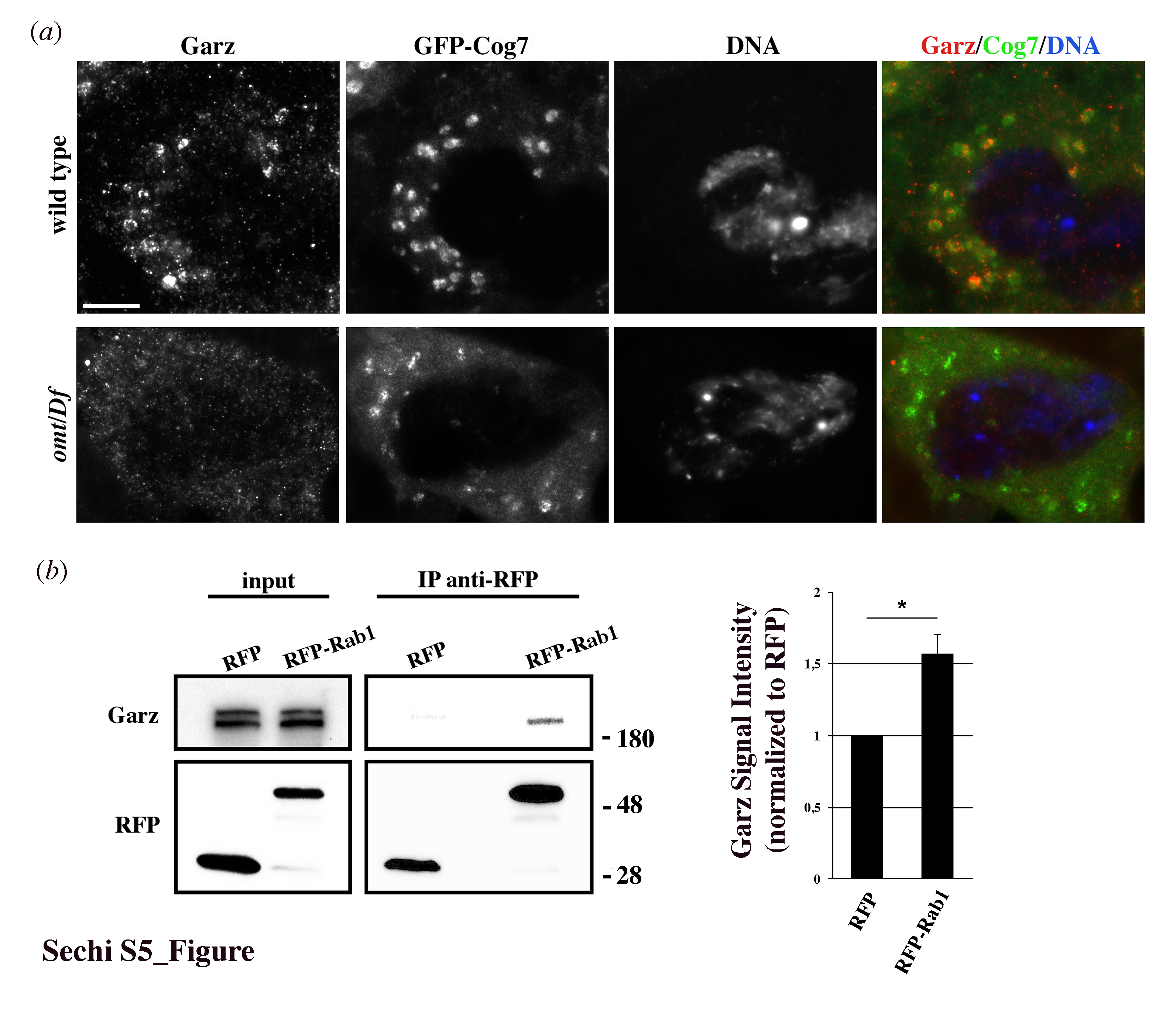

Supplement: Figure S5. Rab1 is required to recruit Garz to the Golgi membranes. [file rsob160257supp5.tif]
